# Prediction of implementing ISO 14031 guidelines using a multilayer perceptron neural network approach

**DOI:** 10.1371/journal.pone.0244029

**Published:** 2021-01-06

**Authors:** Mohamed Mansour, Saleh Alsulamy, Shaik Dawood

**Affiliations:** 1 Industrial Engineering Department, College of Engineering, King Khalid University, Abha, Saudi Arabia; 2 Industrial Engineering Department, College of Engineering, Zagazig University, Zagazig, Egypt; 3 Architecture and Planning Engineering Department, College of Engineering, King Khalid University, Abha, Saudi Arabia; Univerza v Mariboru, SLOVENIA

## Abstract

The purpose of this study was to model the link between the implementation of ISO 14031 and ISO 14001. This study connects ISO 14031’s guidelines as independent variables to a dependent variable expressed by the ISO 14001 certification situation of industrial organizations based on the judgments of environmental managers in Saudi Arabia. Applying the quantitative approach using a survey with 596 responses from organizations functioning in 30 economic activities, a multi-layered neural network was trained to examine the relationship between standards and predict whether the organization is ISO 14001 certified in addition to testing the developed network on a group of collected cases. The results demonstrated the ability of the network to classify the organization’s certification status by 94.00% according to the training sample and its ability to predict 91.00% of the test sample, with an overall prediction efficiency of 91.30%. This work provides insights and adds to the environmental performance evaluation literature providing a neural network model based on ISO 14031 guidelines that can be extended to include other international standards such as ISO 9001. This study supports the merging of ISO 14001 with ISO 14031 into a binding standard.

## Introduction

In recent year, business decision-makers have become interested in investigating the effect of implementation of both environmental management systems (EMSs) and environmental performance evaluation (EPE) given in ISO 14031 as indicated by Falqi, Alsulamy [1]. This interest is due to the befits of implementing EMSs that were categorized into environmental management practices, environmental performance, and awareness [2]. Improved business performance was added as amajor befit by Waxin, Knuteson [3]. A comprehensive literature review about the benefits of ISO 14001 implementation is reviewed by Tarí, Molina-Azorín [4]. ISO 14001 series was first published in 1996 and 307,059 awarded certifications issued related to this standard in 191 countries [5].

The new ISO 14001:2015 edition emphasis on EPE compared with the 2004 edition [6] and its scope, Section 4.4, and Section 5.2 require an organization to evaluate and enhance EPE [7]. In ISO 14001:2015, it is important to follow the guidelines given in ISO 14031:2013 to evaluate and manage the EPE efforts in order to achieve the continuous improvement in environmental indicators [8]. While, EP’s indicators were suggested, categorized, and ranked for measuring the environmental status of an organization [9], environmental reported were suggested for evaluating the EP of an organization [10]. The benefits from adopting ISO 14001 standard were classified by Boiral, Guillaumie [11] as: waste minimization and management, air pollution, EP in general, energy and resources consumption, environmental risks and safety issues, and water contamination respectively based on 94 published article. A conflicting results about ISO 14001 certification and EPE was presented in this article.

The major objective of ISO 14031 is to design and use EP indicators in an organization to determinate the organization's EP [12]. To measure EP, Tóth [13] reviewed 11 methods including eco-mapping, EMS of aspects evaluation, EPE by ISO 14031, eco-efficiency evaluation, eco-balances, environmental performance accounting, multi-step environmental classifications, eco-ratings, environmental performance index, eco-points system, and translations into impacts. Based on surveying the opinions of a sample comprising 370 organizations, Tóth concluded that ISO 14031 is the easiest and most common method used by organizations that use indicators, material and energy balances, and environmental cost accounting for defining the EPE.

Various research has been conducted to investigate the adoption and outcomes of ISO 14001 certification in terms of environmental management practices, EP, and social aspects in Boiral, Guillaumie [14]. Some authors have argued in support of a positive waste minimization/management and ISO 14001 certification association [15, 16] such as in the manufacturing and non-manufacturing industries, Martín-Peña, Díaz-Garrido [17] in the Spanish automotive industry, Schylander and Martinuzzi [18] in Austria, Curkovic and Sroufe [19] in automotive industry companies located in the USA. On the contrary, no significant influence between EP and certification was concluded by Ghisellini and Thurston [20, 21]. No consensus exists among researchers regarding the relationship between air emission/pollution and ISO 14001 certification, positive or no effective relationship. While some authors have argued a significant relationship exists [22, 23], other authors have shown that there is no significant relationship between air emission and certification Aravind and Christmann [24]. Therefore, the nature of this relationship is still unknown and is a subject of further investigation. Some studies have reported no significant improvement with respect to water contamination performance indicators [25, 26]. Conversely, Darnall and Kim [27] found a significant improvement in water contamination performance indicators.

From our literature review, Some studies report mixed results with respect to decreased energy and resource consumption; the standard improved the effectiveness [28, 29] or non-certified firms performed better [30]. A major effect of satisfying ISO 14001 requirements regarding environmental performance is a reduction in environmental risk and safety issues as a result of implementing the standard, as concluded by Zeng, Tam [31], [32]. With respect to the general and aggregate environmental performance of firms, some researchers have noticed a positive significant effect related to the EP to ISO 14001 implementation such as Prajogo, Ailie [33–35]. Other authors found no significant improvement in the environmental performance aggregate indicators related to implementing the standard [36, 37]. These studies have led to conflicting results concerning ISO 14001 certification and EP in terms of single, aggregate, or general indicators. Nonetheless, they have not studied the relationship between implementing the standard and satisfying the requirements of EPE guidelines given in ISO 14031. Previous research has not studied the development of a prediction model concerning managerial efforts and technical environmental indicators. Therefore, the following research questions were posed: “*Are the 596 industrial organizations in Saudi Arabia implementing ISO 14031 guidelines for EPE*?”; “Is there an association between ISO 14001 certification and EPE in Saudi organizations?”; and “*Is it possible to model the relationship between implementing ISO 4031 guidelines and the ISO 14001 certification status of the 596 industrial organizations in Saudi Arabia as a multi-layer neural network*?”

Therefore, the study was conducted on a sample of 596 industrial organizations to analyze the relationship between managerial environmental performance defined by ISO 14031 guidelines and ISO 14001 certification besides developing an artificial neural network (ANN) model as a first trial to measure the overall EP of firms. We have organized the remainder of this paper as follows: Section 2 describes the solution methodology adopted to determine the association between the implementation of ISO 14031 and ISO 14001; Section 3 presents the results of the ANN model in addition section 4 highlights the discussion drawn from the findings; Finally, section 5 presents the conclusions and presents the study limitations and future research avenues.

## Materials and methods

Phi association coefficients, total unduplicated reach and frequency (TURF) analysis, and ANNs were the methods used in this study. Phi coefficients were calculated to measure the association between the standard certification and the requirements of ISO 14031. TURF analysis was used to determine the best reach and frequency by group size, while ANN built the relationship between ISO 14001 certification and ISO 14031 guidelines implementation status in surveyed organizations. The ANN is a computer simulation model that simulates the human brain. The brain included units called neural cells. The cells are connected with each other by connectors. The ANN aims to simulate the functions of the central brain. The ANN model is an excellent approach for building modeling relationships between inputs and outputs of a system with linear and nonlinear complex data sets without the assumptions of an existing relationship between the system inputs and outputs to predict the values of the dependent variable(s) [38]. The multilayer perceptron neural network module in IBM SPSS (version 26.0) was used to build, train, and test the relationship between Y and the independent covariates. The ANN model is a three-layer feed-forward ANN. The network has 13 covariates with a standardized rescaling method. It has a hidden layer with nine neurons, excluding the bias unit. The ANN activation function was selected to be a hyperbolic tangent (HT) in the hidden layer. The output layer of the network has two neurons. SoftMax was used as the activation function. The cross-entropy error function was chosen in the output layer. The IBM SPSS modeler (Version 18) was used to model the current problem as ANNs, C5.0, KNN algorithm, Bayesian network, CHAID, C&R tree, Decision list, and Quest.

### Sample characteristics and data collection

A personnel face to face interviews are conducted with the environmental management engaged personnel’s on their organizations by a representative of the research team. Each interview is lasted between 35 to 45 minutes. The personal interview is accepted and preferred than impersonal interview forms in developing countries and strongly accepted in Saudi Arabia culture [39]. All study participants provided informed consent, and the study design was conducted according to ethical principles for King Khaled University and was approved by Scientific Research Ethics Committee in Industrial Engineering Department (Ethics Committee Date: 01.03.2020, Decision Number: 01-05-42). The participants signed a confidentiality consent form and the interviews included discussion of the questionnaire’ questions, revision of environmental records is adopted as a data verification technique. The most of participants required personal and organization’s anonymity. E-mails were sent and multiple phone calls were made with the candidate organizations to ensure the participation of all certified organizations in the study. The 900 e-mails were sent, of which 317 e-mails were sent to certified organizations in the period of March to April 2020, and responses were received by e-mail in May 2020. It was agreed that the questionnaire respondent should be the manager of the environmental department for each organization. In addition, detailed instructions were included in the questionnaire giving detailed information on how to answer the questions. The questionnaire included a question asking if the organization has ISO 14001:2015 certification, and 13 questions concerning the organization’s commitment to following the guidelines of the ISO 14031 standard to evaluate the environmental performance.

### Participant organizations

There were 317 ISO 14001 certified organizations working in 30 economic activities located in Saudi Arabia until 2018 [40]. Economic activities include construction, forestry, education, and petrochemicals. Consequently, the maximum number of organizations that could be involved in the ISO 14001 certified group for each economic activity was determined from the survey results. As a result, the number of uncertified organizations must not be less than the number of certified organizations. For example, the number of organizations operating in the construction sector equals 46 certified organizations; therefore, 46 organizations were included in the sample, and the number of certified organizations working in the field of engineering services was 25. All 25 organizations were included in the study and so on.

### Model variables, dataset partition, and architecture design

The input vector to the ANN specifies the 13 independent variables representing the main guidelines in ISO 14031:2013. The variables could be grouped into three main dimensions. The first dimension is related to EPE’s planning action taken by organizations to ensure that planning for EPE will be implemented to measure performance indicators. The second dimension includes the processes and procedures implemented to design, analyze, and implement data and information related to EPE. The third dimension specifies the auditing and improvement of EPE efforts in the organization. Definitions of the 13 variables and the study’s questionnaire are given in Falqi, Alsulamy [1]. Each question is a binary variable answered by “yes” or “no”. The value of “yes” was represented by “1” and the value of “no” was represented by “0” in the ANN model. The organization certification status is a binary variable. The values 0 and 1 indicate ISO 14001 classified and certified organizations. To implement ANN, the dataset was divided into three groups: training, testing, and holdout. The random cases assigned were based on the relative number of cases, and the dataset was partitioned by default in SPSS into a 7:3:0 ratio. The training group contained training data records, and the test group contained data records used to track errors and used a holdout group to evaluate the network architecture efficiency.

Automatic architecture selection was chosen for the design of the ANN with a "feed forward architecture" in which the network can contain two hidden layers as a maximum. In this research, the network was created using a hidden layer. The number of hidden layer units was calculated by default in SPSS using 1 and 50 as the minimum and maximum units. The HT and Softmax activation functions were used to relate the weighted sum of two consecutive layer units. The HT function converts real values to the range (-1,1), while Softmax converts the real values to the range (0, 1). In the article, the dependent variable is of a categorical nature and therefore there is no need to specify a "rescaling method". No feedback loops were used in the forward feed structure of the developed network. The input layer contained five “P” predictors for the first dimension, six “D” predictors for the second dimension, and two predictors (R1 and I1) for the third dimension. The hidden layer also contained hidden nodes that express some predictor functions. The output layer contained responses and was re-coded as two output variables.

### ANN training, output, and stopping rule

Adebiyi, Adewumi [41] confirmed that ANN training is a major factor of success for the network to accomplish its purpose. For the dataset in this research, batch training was defined by default to update the synaptic weights. The scaled conjugate gradient method was automatically selected, and training options such as initial λ, initial σ, interval center, and interval offset did not need to be changed. The optimization training options were adjusted by default to be 5e-7, 5e-5, 0, and ±0.5 as initial λ, initial σ, interval center, and interval offset, respectively. The ANN output was characterized by the network structure and performance. The structure was given by a description, diagram and the values of synaptic weights. The network performance is measured by model summary, classification results, ROC curve, gains chart, case processing summary, and independent variables importance analysis in IBM SPSS.

The ANN algorithm’s stopping rules were adjusted to perform in the following sequence with adjusted values. a) Exclude dependent and independent variables’ missing values. b) A step without a decrease in error and automatic use of the test sample for computing the prediction error. If the test sample did not exist, the training sample was used to compute the prediction error. c) The maximum training time was adjusted to 15 min. d) SPSS was adjusted to automatically calculate the maximum number of training data passes. e) SPSS default values of 1.0e-4, 1.0e-3, and 1000 were set for the minimum relative change in training error, the minimum relative change in training error ratio, and maximum cases to store in memory, respectively. Before running the ANN, the random number generation module in SPSS under the “Transform” menu allowed us to define a random seed. This allowed us to replicate the results of the neural network exactly, irrespective of providing training several times. A fixed value of 2000000 was used for the random number generator seed. Experiments were conducting on an Inspiron 15–3567 Dell laptop, Intel® Core™ i7-7500U CPU@ 2.70GHz (4 CPUs), 2.9GHz, and 8192MB RAM of memory functioning by Windows 10 Enterprise N 2016 LTSB 64-bit (10.0, Build 14393).

## Results

**Fig 1:**
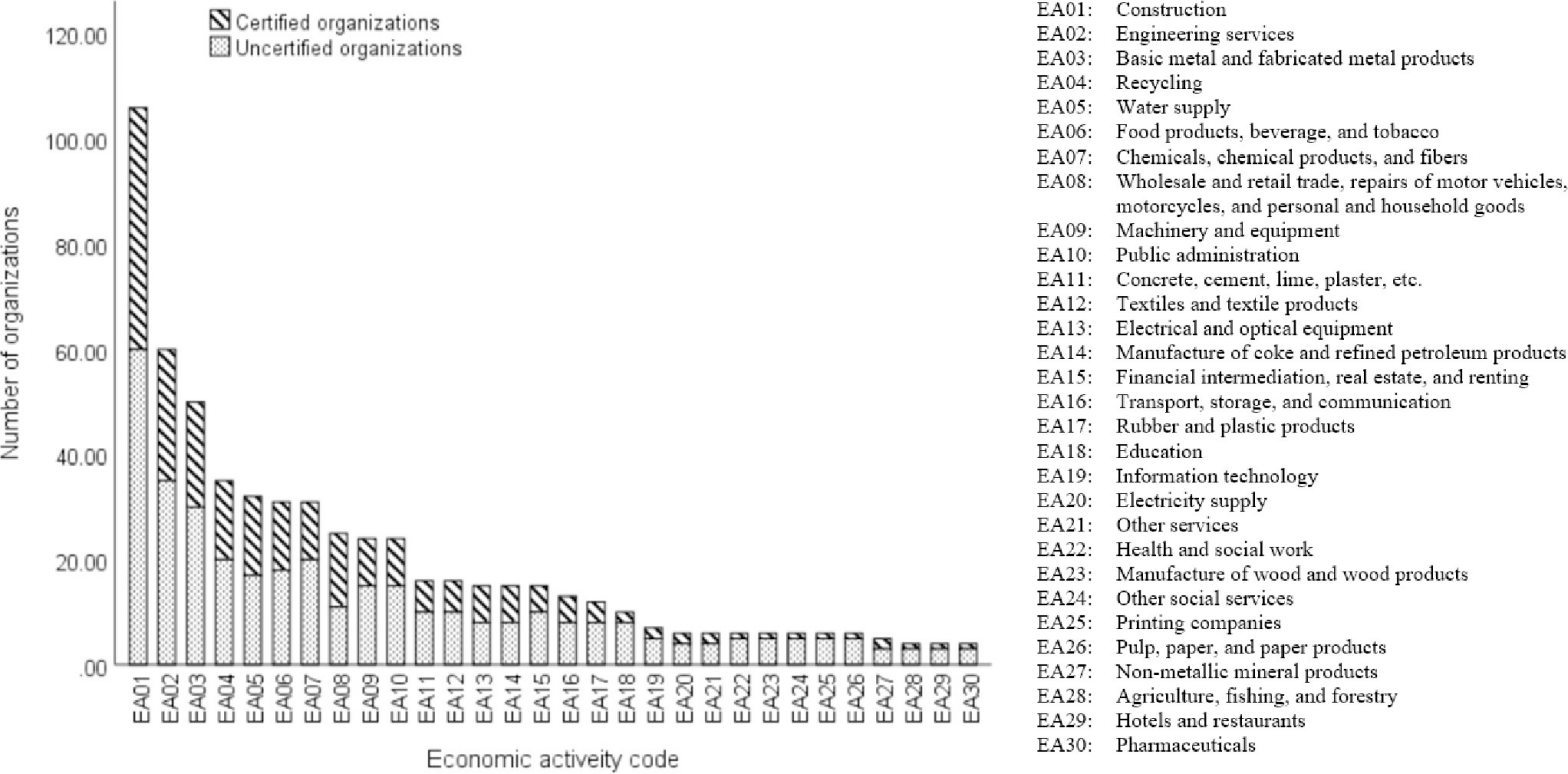
Responses of certified and uncertified organizations and their industrial fields.

The response rate of the questionnaire was 66.22% from 900 emailed organizations, of which 39.43% of responses are ISO 14001 certified while 60.57% are uncertified organizations. The 36.24% of organizations which responded were classified in construction, engineering services, and metal economic activities. Fig 1 depicts a bar chart for the number of certified, uncertified, and industrial fields of the responding organizations. The 30 economic activities are coded from EA01 to EA30. For example, EA01 codes the construction activity and EA02 codes engineering service activities. Of the organizations that responded, 62.08% were categorized in 7 economic activities, namely construction (EA01); engineering services (EA02); metal and fabricated metal products (EA03); recycling (EA04); water supply (EA05); food products, beverages, and tobacco EA(06); and chemicals, chemical products, and fibers (EA07). The industrial sectors that participated in the survey the least were agriculture/fishing-forestry (EA28), hotels and restaurants (EA29), and pharmaceuticals organizations (EA30).

Table 1 summarizes the responses to each ISO 14031 guideline. Of the respondents, 55.9% said they implemented the standard guidelines and 44.1% said they did not, showing a somewhat favorable response. The P4 and R1 guidelines are the most implemented guidelines with, 58.1% of respondents implementing then, and the lowest implemented guideline is P3 at 53.4%. It is evident from the table that regardless of ISO 14001 certification, the respondents generally implement the ISO 14031 guidelines to measure the organization’s environmental performance.

**Table 1:** Frequency table for ISO 14031 guidelines from respondents.

ISO 14031 guideline code	Frequency		Frequency percent	
	0	1	0	1
P1	323	273	54.2	45.8
P2	340	256	57.0	43.0
P3	337	259	56.5	43.5
P4	346	250	58.1	41.9
P5	329	267	55.2	44.8
D1	338	258	56.7	43.3
D2	328	268	55.0	45.0
D3	318	278	53.4	46.6
D4	344	252	57.5	42.3
D5	327	269	54.9	45.1
D6	331	265	55.5	44.5
R1	346	250	58.1	41.9
I1	325	271	54.5	45.5
Average			55.9	44.1

### a) TURF analysis

The TURF analysis module in SPSS was conducted to measure the best reach and frequency by group sizes ranging from 1 to 14, as shown in Table 2. For example, the D3 was added by the SPSS TURF algorithm as the best reach if the group size equals 1 and the pairs (D5, P1) were the most nominated group implemented together with a reach of 392 out of 596 organizations, representing 65.8% implementation. The best group is illustrated in the table as the fourteenth group, which includes all ISO 14031 guidelines from P1 to I1 in addition to the variable Y that corresponds to the implementation status of the organizations with 95.5% number of cases out of 596 cases. This indicates that 95.5% of ISO 14001 certified organizations implement the guidelines for EPE given in ISO 14031.

**Table 2:** Best reach and frequency by group size.

Variables	Statistics				
	Group Size	Reach	% of Cases	Frequency	% of Responses
D3	1	278	46.6	278	7.6
D5, P1	2	392	65.8	542	14.8
D4, D5, P1	3	458	76.8	794	21.7
D2, P3, P4, R1	4	500	83.9	1027	28.1
D3, D2, P3, P4, R1	5	521	87.4	1305	35.7
P1, D2, D3, P3, P4, R1	6	536	89.9	1578	43.2
D5, D2, D3, P1, P3, P4, R1	7	547	91.8	1847	50.6
D4, D6, I1, D2, D5, P1, P3, R1	8	554	93.0	2107	57.7
D1, P2, P4, D5, D6, I1, P1, P3, R1	9	560	94.0	2351	64.4
P5, D1, D5, D6, I1, P1, P2, P3, P4, R1	10	565	94.8	2618	71.7
D1, D5, D6, I1, P1, P2, P3, P4, P5, R1	11	567	95.1	2896	79.3
D2, D1, D3, D5, D6, I1, P1, P2, P3, P4, P5, R1	12	568	95.3	3164	86.7
D4, D1, D2, D3, D5, D6, I1, P1, P2, P3, P4, P5, R1	13	569	95.5	3416	93.6
*Y*, D1, D2, D3, D4, D5, D6, I1, P1, P2, P3, P4, P5, R1	14	569	95.5	3651	100.0

### b) ISO 14031-ISO 14001 association significance and measure

To test the independence of implementing ISO 14031:2013 guidelines by industrial organizations, the chi-square test of significance was used at a 0.05 level of significance. In SPSS, analysis, descriptive statistics procedure testing the independence of *Y* with respect to the 13 independent variables yielded a p-value equal to 1e-3, indicating the significance of the relationship between the independent and independent variables. To test the degree of association among variables, Phi association coefficients were calculated, as shown in Table 3. The value of Phi indicates that there is a medium positive association between Y and the 13 “P”, “D”, R1”, and “I1” variables. The Phi values of association of Y with P5, P1, D2, I1, D6, D3, P2, D1, P3, R1, D4, D5, and P4 arranged from largest to smallest are equal to 0.537, 0.505, 0.465, 0.463, 0.460, 0.450, 0.430, 0.418, 0.415, 0.413, 0.401, 0.393, and 0.358 respectively. The sign “*” indicates significance at p<0.05, as shown in Table 1.

**Table 3:** Phi association coefficients between ISO 14001 certification status and ISO 14031 guidelines implementation.

ISO 14031 measurement variables	Phi correlation coefficient
P1	.505*
P2	.430*
P3	.415*
P4	.358*
P5	.537*
D1	.418*
D2	.465*
D3	.450*
D4	.401*
D5	.393*
D6	.460*
R1	.413*
I1	.463*

### c) ANN model

By running the multilayer perceptron module in SPSS under the neural network-analyze module, we obtained the case processing summary as shown in Table 4. The table shows that 401 cases representing 67.3% of the total respondent organizations were allotted for the training sample, while 195 cases were allotted for testing the developed neural network. All data were included in the neural network building process, and no data were excluded.

**Table 4:** Case processing summary.

		N	Percent
Sample	Training	401	67.3%
	Testing	195	32.7%
Valid		596	100.0%
Excluded		0	
Total		596	

Table 5 presents the ANN model summary. The network training was characterized by a cross-entropy error during training equal to 61.008, and a cross entropy error of 40.328 in testing, 6.0% of incorrect training predictions, and 8.7% of incorrect testing predictions, respectively. The training stopped using one consecutive step with no decrease in error, whereas error computations were based on the testing sample. 0.14 s was consumed in training the network.

**Table 5:** ANN’s model summary.

Training	Cross entropy error	61.008
	Percent incorrect predictions	6.0%
	Stopping rule used	1 consecutive step with no decrease in error
	Training time	0.14 seconds
Testing	Cross entropy error	40.328
	Percent incorrect predictions	8.7%

**Fig 2:**
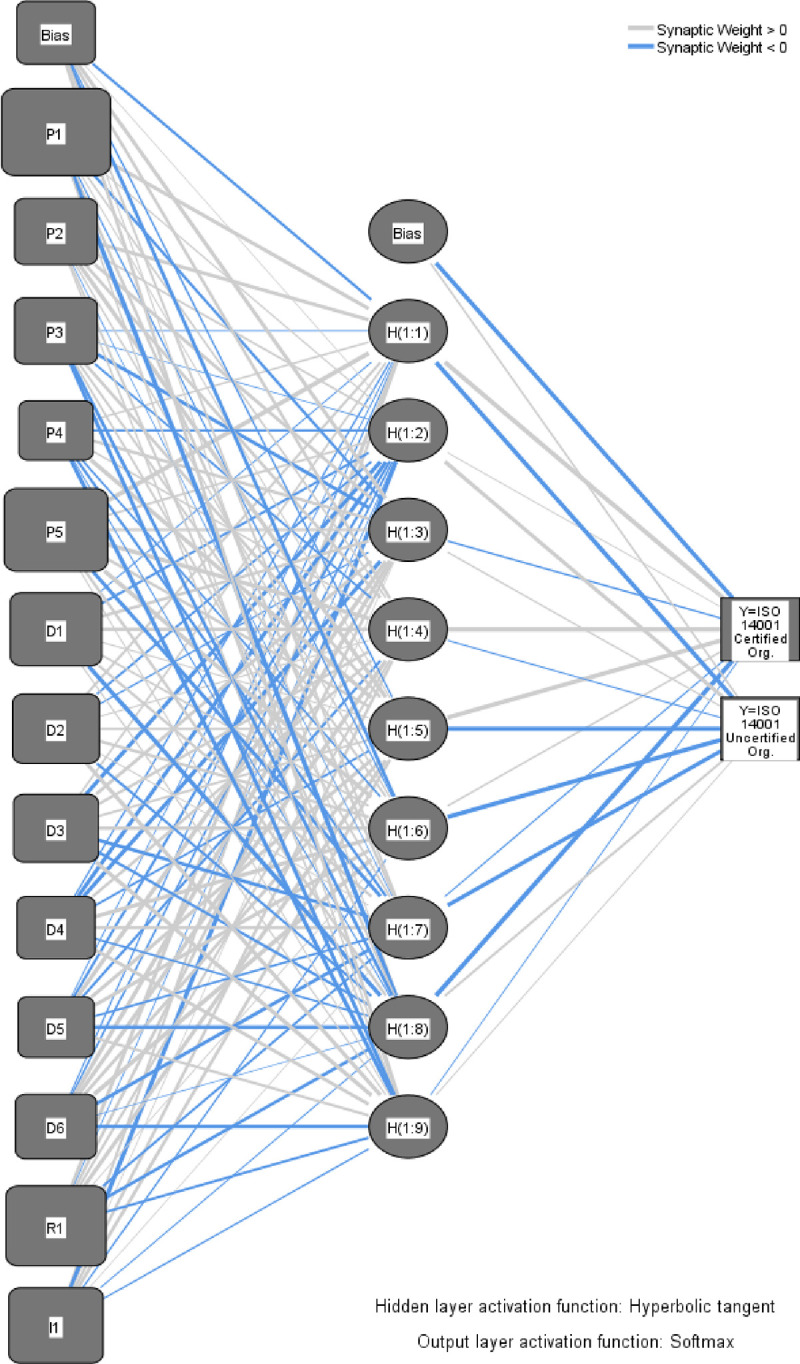
The multilayer perceptron ANN’s diagram.

Evaluation of the strength of the relationship between ISO 14031 guidelines in terms of Ps, Ds, R1, and I1 with ISO 14001 certification was performed in a two-stage process based on the parameter values for the developed ANN architecture. The developed ANN is shown in Fig 2. Parameter estimates in terms of weights and bias for hidden layer 1 and output layer parameter estimates for the output layer are given in Table 6. H(i:j) denotes the hidden layer i and j^th^ neuron. The weight attached to H(1:1) neuron from the Bias node is -0.267, 0.417 from P1, 0.408 from P2, -0.037 from P3, 0.133 from P4, 0.674 from P5, -0.055 from D1, is 0.243 from D2, 0.151 from D3, -0.111 from D4, -0.046 from D5, -0.096 from D6, is 0.418 from R1, and 0.261 from I1 and so on for other neurons in the input layer. The weights from hidden layer 1 to the output layer (ISO 14001 uncertified Org. and ISO 14001 certified Org.) neurons for bias are -0.531 and 0.136, respectively. The weights for the first neuron in the hidden layer 1 H(1:1) to *Y* = 1 and *Y* = 0 are 0.678 and -0.595, respectively.

**Table 6:** Parameter estimates—hidden layer 1 and output layer.

Predictor		Predicted hidden layer 1									Predictor		Predicted output layer (*Y*)	
		H(1:1)	H(1:2)	H(1:3)	H(1:4)	H(1:5)	H(1:6)	H(1:7)	H(1:8)	H(1:9)			ISO 14001 uncertified Org.	ISO 14001 certified Org.
Input layer	(Bias)	-.267	.000	.422	.327	-.115	-.377	.184	.076	.357	Hidden layer 1	(Bias)	-.531	.136
	P1	.417	.090	-.281	.137	.072	-.091	.318	-.654	.122		H(1:1)	.678	-.595
	P2	.408	.157	.365	.331	.463	.120	-.017	.215	.123		H(1:2)	.044	.427
	P3	-.037	-.037	-.400	-.161	.351	.186	.160	-.361	-.416		H(1:3)	-.122	.106
	P4	.133	-.162	.352	.135	.527	-.144	-.262	-.058	-.442		H(1:4)	.713	-.077
	P5	.674	.184	.320	.537	.296	.590	-.410	.294	.064		H(1:5)	.612	-.629
	D1	-.055	-.233	.342	.374	.080	.153	.281	-.542	.097		H(1:6)	.131	-.648
	D2	.243	-.184	-.105	.085	.173	.323	.050	-.256	.439		H(1:7)	-.087	-.449
	D3	.151	-.428	.363	.287	.192	.405	-.392	-.276	.460		H(1:8)	-.878	.209
	D4	-.111	-.427	-.435	.304	-.166	.444	.354	-.144	.368		H(1:9)	-.041	.052
	D5	-.046	-.258	.492	-.258	.405	.411	-.169	-.364	.271				
	D6	-.096	-.14	.472	.471	.362	.608	-.385	-.005	-.409				
	R1	.418	-.039	.329	.249	.442	.040	-.198	-.354	-.256				
	I1	.261	-.592	.021	.329	.361	-.109	.010	-.064	-.106				

Table 7 presents the classification table for the independent variable *Y*. The overall percentage of correct predictions in the training sample was 94.0%. The percentage of incorrect predictions was 4.0%, which is calculated as 100% subtracted from 94%. Likewise, the percentage of incorrect predictions in the testing sample was 8.7% because the percent of correct predictions was 91.3%. The area under curve (AUC) value for the developed ANN was 0.983 for predicting *Y* = 1 and *Y* = 0. This value indicates that the ANN has an excellent ability to predict or classify *Y* [42].

**Table 7:** Classification table-dependent variable (*Y*).

Sample	Observed	Predicted		
		ISO 14001 certified Org.	ISO 14001 uncertified Org.	Percent correct
Training	ISO 14001 certified Org.	149	13	92.0%
	ISO 14001 uncertified Org.	11	228	95.4%
	Overall Percent	39.9%	60.1%	94.0%
Testing	ISO 14001 certified Org.	67	6	91.8%
	ISO 14001 uncertified Org.	11	111	91.0%
	Overall percent	40.0%	60.0%	91.3%

**Fig 3:**
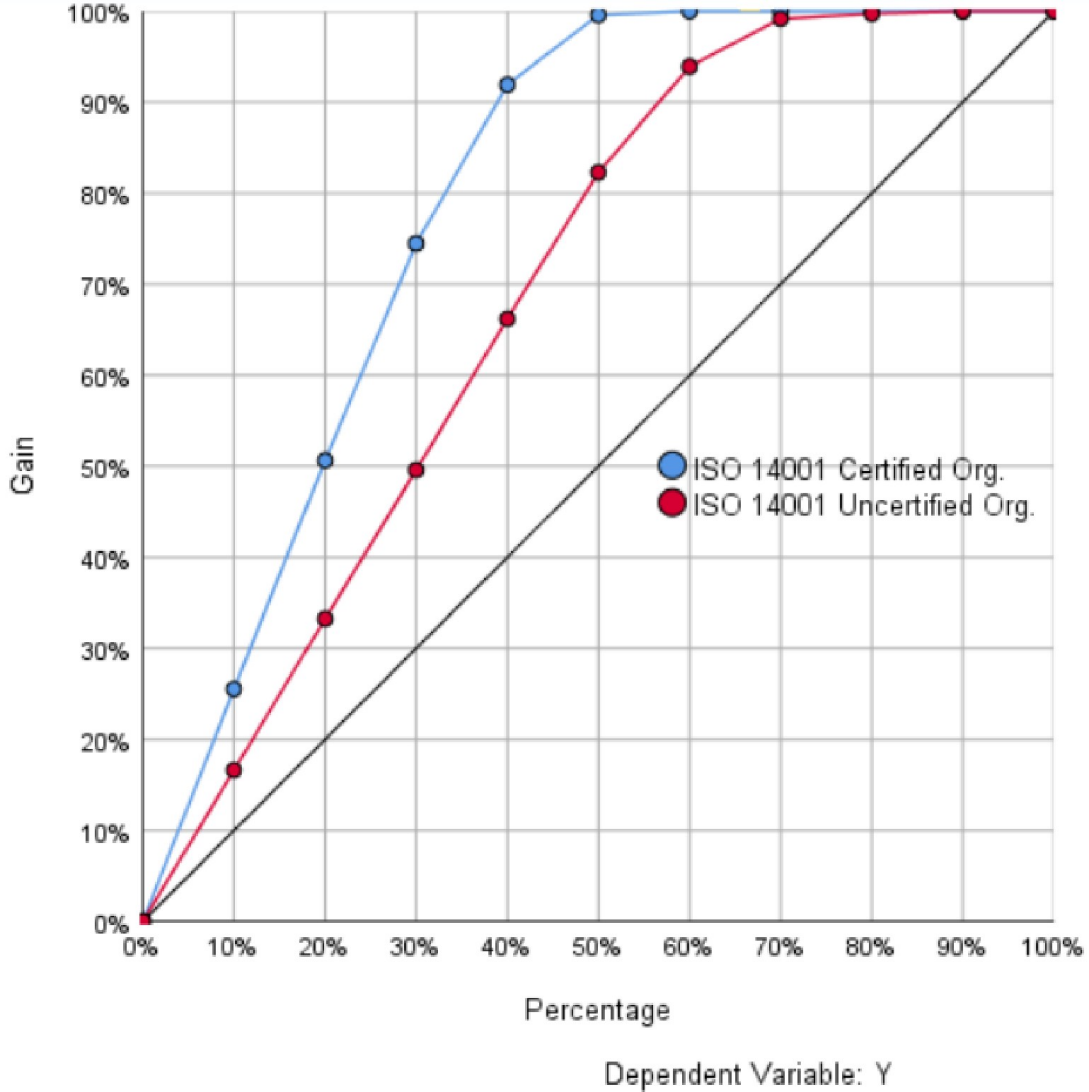
Cumulative gain chart.

**Fig 4:**
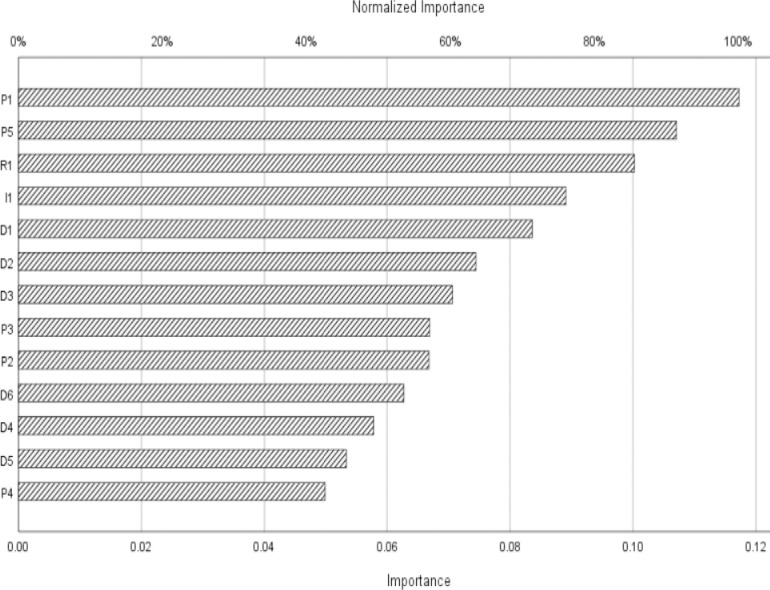
Independent variable importance chart.

Fig 3 shows the cumulative gain chart for the developed ANN. This chart displays the ratio of the total number of cases earned by each category with respect to the total number of cases. It can be seen from the chart that the first point of the category 1 curve (in blue) is in the gain interval (20%, 30%), which means that more than 10% of cases contain approximately 25% of cases that actually take the value 1; likewise, 20% of cases contain approximately 50% and so on, until 100% of cases and return are reached. Because all points are higher than the baseline curve, the prediction performance of the network for cases that result 1 and cases that result 0 is satisfactory. Fig 4 shows the normalized importance of each predictor variable arranged in descending order. As the figure depicts; the normalized importance percentages of P1, P5, R1, I1, D1, D2, D3, P3, P2, D6, D4, D5, and P4 were 100.00, 91.30, 85.50, 76.00, 71.30, 63.50, 60.30, 57.10, 57.00, 53.50, 49.30, 45.50, and 42.60 respectively.

### d) Performance evaluation of the developed ANN

Different experiments with different random seeds were conducted to determine whether the developed ANN’ results were random. Fifty nine simulation run were conducted to estimate the overall percent correct and the consumed CPU time from running the developed ANN as listed in Table 8. The algorithm overall percent correct was 92.30 paired with an average CPU time of 0.14 seconds. The range for overall percent correct was 11.10 (minimum value = 8.80 and maximum value = 97.90), while the CPU time rage was 0.27 (minimum CPU time = 0.01 seconds and maximum CPU time = 0.28 seconds). Shapiro-Wilk p test was conducted for normality checking of both response variables. The values of 0.06 and 0.08 as shown in the last row of Table 8, indicated that both the overall percent correct and CPU time were normal.

**Table 8:** Descriptive statistics for overall percent correct and CPU run times for the developed ANN.

	Overall percent correct	CPU run times
Sample size	59.00	59.00
Mean	92.30	0.14
Minimum	86.80	0.01
Maximum	97.90	0.28
Shapiro-Wilk p	0.06	0.08

### e) Experimental comparison of the developed ANN model with other algorithms

**Fig 5:**
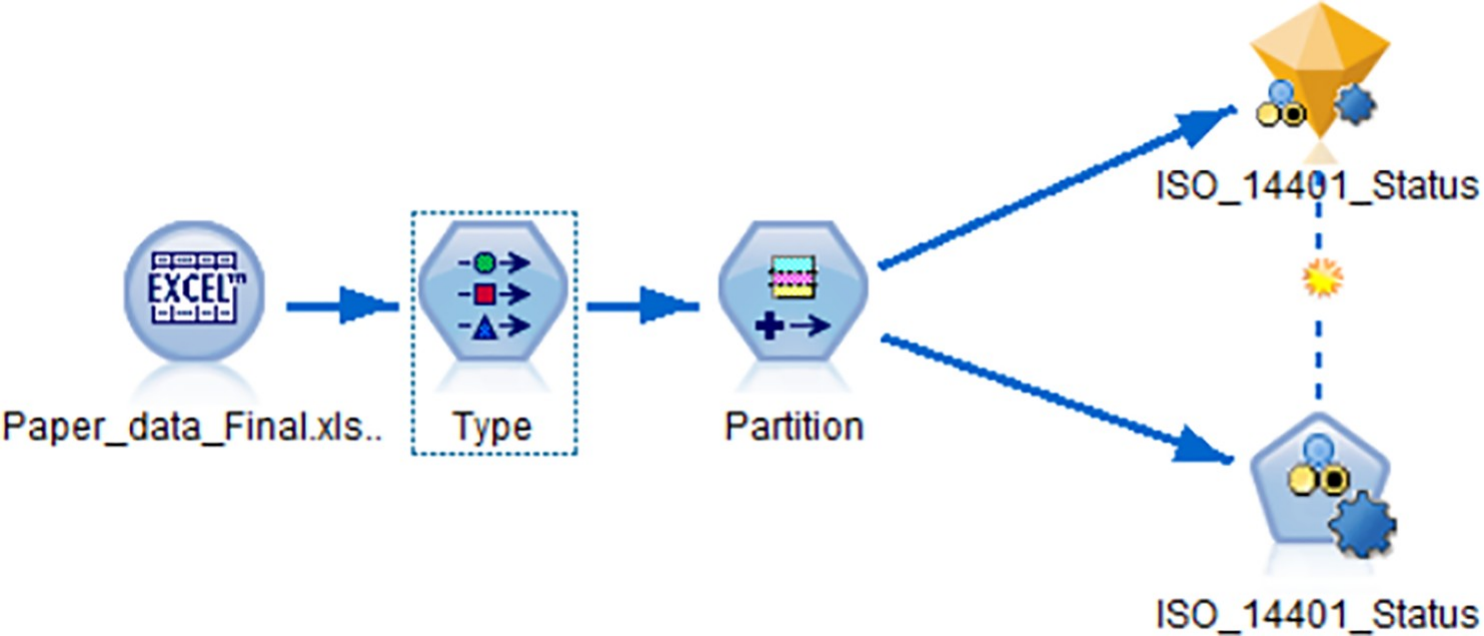
IBM SPSS modeler's model structure.

It is recommended to use updated methods such as other neural networks or other methods for experimental comparison to ensure the reliability of the results. The comparative models were built using IBM SPSS Modeler data mining workbench for building accurate predictive models. The auto classifier node in SPSS modeler was used to create and compares 8 models for binary decision analysis. The comparative models were ANNs, C5.0, KNN algorithm, Bayesian network, CHAID, C&R tree, Decision list, and Quest. The C5.0 builds either a decision tree or a rule set. The k-Nearest Neighbor (KNN) associates a new case with the category or value of the k objects nearest to it in the predictor space, where k is an integer. The Bayesian network node enables a probability model to establish the likelihood of occurrences. The node focuses on Tree Augmented Naïve Bayes (TAN) and Markov Blanket networks for classification. The CHAID algorithm generates decision trees using chi-square statistics to identify optimal splits. The Classification and Regression (C&R) Tree algorithm generates a decision tree that allows predicting or classifying future observations. The decision list model identifies subgroups, or segments, that show a higher or lower likelihood of a given binary outcome relative to the overall population. The QUEST model provides a binary classification method for building decision trees. The developed model was shown in Fig 5. The 8 models were compared based on max profit, max profit occurs in (%), lift (top 30%), overall accuracy (%), and area under curve as shown in Table 9.

**Table 9:** Comparing and ranking different modeling approaches with ANNs.

Model	Max profit	Max profit occurs in (%)	Lift (top 30%)	Overall accuracy (%)	Area under curve
Neural net	300.00	38.00	2.31	91.11	0.98
C5.0	205.00	42.00	1.88	81.11	0.84
KNN algorithm	298.08	43.00	2.31	88.89	0.96
Bayesian network	295.00	42.00	2.31	90.00	0.97
CHAID	223.33	50.00	2.02	80.00	0.88
C&R tree	230.00	42.00	2.03	80.56	0.88
Decision list	205.00	32.00	2.05	76.67	0.87
Quest	134.87	27.00	1.80	73.33	0.76

Results summarized in Table 9 indicated the superiority of ANNs as a modeling approach for predicting the status of a company regarding the compliance for ISO 14001 requirements. ANN’s model achieved 300.00, 38.00, 2.31, 91.11, and 0.98 for max profit, max profit occurs in (%), lift (top 30%), overall accuracy (%), and area under curve respectively. The C5.0 model achieved a performance of 205.00, 42.00, 1.88, 81.11, and 0.84 for max profit, max profit occurs in (%), lift (top 30%), overall accuracy (%), and area under curve respectively. KNN algorithm achieved a performance of 298.08, 43.00, 2.31, 88.89, and 0.96 for max profit, max profit occurs in (%), lift (top 30%), overall accuracy (%), and area under curve respectively. Bayesian network model achieved a performance of 295.00, 42.00, 2.31, 90.00, and 0.97 for max profit, max profit occurs in (%), lift (top 30%), overall accuracy (%), and area under curve respectively. CHAID model achieved a performance of 223.33, 50.00, 2.02, 80.00, and 0.88 for max profit, max profit occurs in (%), lift (top 30%), overall accuracy (%), and area under curve respectively. C&R tree achieved a performance of 230.00, 42.00, 2.03, 80.56, and 0.88 for max profit, max profit occurs in (%), lift (top 30%), overall accuracy (%), and area under curve respectively. Decision list model achieved a performance of 205.00, 32.00, 2.05, 76.67, and 0.87 for max profit, max profit occurs in (%), lift (top 30%), overall accuracy (%), and area under curve respectively. Quest model achieved a performance of 134.87, 27.00, 1.80, 73.33, and 0.76 for max profit, max profit occurs in (%), lift (top 30%), overall accuracy (%), and area under curve respectively.

## Discussion

The objective of this article is to investigate the link between ISO 14001 certification and the implementation of the non-binding ISO 14031 guidelines. Due to disagreement among researchers about ISO 14001 certification and the environmental performance of organizations [22, 23, 25], and the lack of research comparing the link on an administrative basis, this survey study aimed to investigate the research gap with ISO14031 guidelines. The EPE was measured based on executing the guidelines for EP given in ISO 14031 by 596 Saudi industrial organizations working in 30 economic activities during the period from March to April 2020. To attain the objective, the study focused on answering three questions.

First, “What is the extent of commitment from ISO 14001 certified organizations to apply the non-binding ISO 14031 guidelines for assessing environmental performance in the Saudi Arabia?” Our descriptive analysis revealed that the implementation of ISO 14031 guidelines ranged from 41.9 to 45.8%, with an average of 44.1%. This is consistent with the results by Mansour, Khadar [43] regarding the importance of implementing environmental laws in Saudi Arabian organizations in the construction industry. Second, “Is there an association between ISO 14001 certification and EPE in Saudi organizations?” Our analysis in terms of measuring the association between the two standards and TURF analysis revealed significant associations at a significance level of 0.05, with the largest association coefficient of 0.537 for P5 and a minimum of 0.358 for P4. In addition, it was revealed from the TURF analysis that 95.5% of ISO 14001 certified organization responses (235 organizations) agree with the implementation of all ISO 14031 guidelines. This result coincides with previous research supporting the existence of a positive relationship between the two standards [15–17, 19]. Our results contradict the hypothesis that there is no association between EPE and ISO 14001 certification reported in [11]. Third, “Is it possible to model the relationship between implementing ISO 4031 guidelines and the ISO 14001 certification status of 596 industrial organizations in Saudi Arabia as a multi-layer neural network?” The developed ANN model can be used to classify industrial organizations as those that do and those that do not have an EMS. The developed model can be coded by a computer program and used by officials and representatives of municipalities to estimate the extent of organization’s conformity to EMSs, regardless of their ISO 14001 certification status. The overall prediction efficiency of the developed ANN was 91.30%. The ANN model was compared with other 7 modeling approaches to evaluate the reliability of results. The 7 models were C5.0, KNN algorithm, Bayesian network, CHAID, C&R tree, Decision list, and Quest. ANNs outperformed other 9 models with a performance of 300.00, 38.00, 2.31, 91.11, and 0.98 for max profit, max profit occurs in (%), lift (top 30%), overall accuracy (%), and area under curve respectively.

This research highlights a basis for building a prediction model to measure the sensitivity of each influencing variable in ISO certification using ANN. The study creates a path to measure and evaluate the sensitivity and performance of each industry type by including the type of economic activity in the prediction model. The flexibility of the neural network approach enables investigators and practitioners to access and include many performance indicators in the model. Although the developed ANN model represents a first step in relation to ISO 14001 and ISO 14031, considerable additional research needs to be conducted. For example, the developed model concentrates on the main ISO 14031 clauses and requirements such as management, operation, and general environmental condition indicators, and further research may be required to include engineering and technical indicators as a part of the overall prediction model. Moreover, a limitation arises related to the time of ISO 14001 certification, the period the company has been certified, and the stability of environmental manpower in working in the same company. Also, the different industries were not modeled as independent variable the developed ANN model. These factors may change the prediction model behavior and can be included in future research.

Our study has implications because it studies the link between ISO 14001 certification and ISO 14031 guidelines implementation in Saudi industries. Each independent variable may represent a collection of sub-variables related to major ISO 14031 guidelines. For example, the EPE variable may include indicators for measuring the managerial efforts, technical/engineering indicators such as air pollution, the percentage of heavy particles in water, and performance indicators. Increasing the variables in our model may change the results and accuracy of the developed ANN. This study is beneficial to industrial sectors, particularly to general, large societies. Thus, they can grasp the guidelines of the ISO 14031 standard and adopt ISO 14001 certification, which enhances their utilization ability for marketing and development. Moreover, customers and government officials will be able to easily analyze EMSs of organizations. The added benefit from the ISO 14001 developed model is that it creates an importance of the critical factors that influence EPE by the value of coefficients, and it enhances field experts to perform perfect environmental planning by inserting variables to create more comprehensive measurable factors.

## Conclusions

The article’s purpose is to investigate whether ISO 14001 certified organizations use the guidelines in the international standard ISO 14031 to evaluate their environmental performance. The reviewed research did not reach a unique relationship between EPE and EMS implementation, such as ISO 14001 or EMAS. Some researchers have argued that a positive, negative, or zero relationship associates both EPE and EMSs in various economic activities. Overall, the findings suggest a positive association between implementing ISO 14031 guidelines and implementing the EMS ISO 14001 based on analyzing and modeling the responses of 596 industrial organizations ISO 14031 implementation. The proposed ANN modeling approach is a flexible approach for relating the independent variables related to EPE efforts to ISO 14001 implementation.

In this article, we used subjective questionnaires to record the status of implementing the guidelines using a binary response. Future research may be required to conduct field audits by independent lead auditors for industrial organizations in different countries. In addition, in this article we used general EPE guidelines in ISO 14031 regardless of the type of economic activity of the organization. Furthermore, additional research is required to construct a way to enable organizations in the same field to benchmark their managerial procedures and activities in a consistent way to measure environmental performance. Using a binary scale limited this research in using various statistical analysis and modeling techniques, further research may be valuable to incorporate various types of Likert scales and other modeling techniques such as discriminant analysis, linear, logistic, and Cox regression, generalized linear mixed model, and clustering models for a comprehensive analysis.
